# Attitude Algorithm of Gyroscope-Free Strapdown Inertial Navigation System Using Kalman Filter

**DOI:** 10.3390/mi15030346

**Published:** 2024-02-29

**Authors:** Xiong Jiang, Tao Liu, Jie Duan, Maosheng Hou

**Affiliations:** School of Opto-Electronic Engineering, Changchun University of Science and Technology, Changchun 130022, China; 13251812435@163.com (X.J.); liutao@cust.edu.cn (T.L.); houmsh@cust.edu.cn (M.H.)

**Keywords:** attitude algorithm, accelerometer array, angular velocity calculation, Kalman filtering

## Abstract

A gyroscope-free strapdown inertial navigation system (GFSINS) solves the carrier attitude through the reasonable spatial combination of accelerometers, with a particular focus on the precision of angular velocity calculation. This paper conducts an analysis of a twelve-accelerometer configuration scheme and proposes an angular velocity fusion algorithm based on the Kalman filter. To address the sign misjudgment issue that may arise when calculating angular velocity using the extraction algorithm, a sliding window correction method is introduced to enhance the accuracy of angular velocity calculation. Additionally, the data from the integral algorithm and the data from the improved extraction algorithm are fused using Kalman filtering to obtain the optimal estimation of angular velocity. Simulation results demonstrate that this algorithm significantly reduces the maximum value and standard deviation of angular velocity error by one order of magnitude compared to existing algorithms. Experimental results indicate that the algorithm’s calculated angle exhibits an average difference of less than 0.5° compared to the angle measured by the laser tracker. This level of accuracy meets the requirements for attitude measurement in the laser scanning projection system.

## 1. Introduction

Laser scanning projection systems are widely used in advanced intelligent manufacturing. These systems utilize the 3D CAD model of parts and employ high-precision dual-axis scanning galvanometers to rapidly deflect laser beams, projecting contour frames onto the workpiece [1]. However, during practical applications, vibrations and shock loads can cause the projection system’s attitude to deviate, resulting in inaccuracies in guiding part installation and positioning. To ensure precise assembly, secondary calibration is necessary. This involves re-establishing the coordinate transformation relationship between the laser scanning projection system and the projected object. Accurately determining the attitude change in the laser scanning projection system becomes a key challenge in achieving high-precision guidance for mounting and positioning. Many studies have been conducted to address the problem of attitude change in instruments subjected to vibration and shock loads [2,3,4,5,6,7]. Jiao et al. have explored the use of accelerometer array testing technology in vibration environments. This technology effectively measures the absolute impact of coupling effects caused by multi-degree-of-freedom linear and angular vibrations [8]. Accelerometer array technology not only possesses the autonomy characteristics found in inertial navigation systems but also overcomes their complexity, high cost, and maintenance difficulties.

Development efforts for accelerometer arrays have been going on for over 30 years. The major motivation for the investigation of the GFSINS is that gyroscopes with high precision and high reliability are usually costly, and a conventional inertial measurement unit equipped with lower-cost gyroscopes often suffers from the drawbacks of gyroscopes, such as large bias instability [9,10]. However, the use of an array composed of low-cost accelerometers can improve this. The current research mainly focuses on the accelerometer configuration schemes in various application areas and the corresponding calculation algorithms for angular velocity. One of the key issues in GFSINS research is improving the accuracy of the angular velocity calculation. M.C. Algrain’s theoretical work established that a minimum of six linear accelerometers is necessary to fully capture the motion of a rigid body in an entirely independent GFSINS [11]. Building on this foundation, Chen developed an innovative arrangement for these six accelerometers by positioning one on each face of a cube, oriented towards the diagonals, with the measurement system’s origin at the cube’s center [12]. This arrangement, known as the cubic configuration, has become the standard for deploying six accelerometers. Despite its simplicity, this approach for deducing angular velocity through the integration of diagonal accelerations is prone to accumulating substantial errors. Jose adopted a cubic configuration based on linear optomechanical accelerometers. The results show the precision when considering the accelerometer’s thermal noise limit can be orders of magnitude better than MEMS systems and comparable to fiber optical gyroscopes [13]. Ma initially proposed a redundant configuration scheme involving nine accelerometers to correct angular velocity [14]. While experimental evidence has shown that this approach effectively suppresses the divergence of angular velocity errors, the design necessitates a high level of installation precision at the centroid, making it challenging to implement in engineering applications.

Researchers have discovered that employing a twelve-accelerometer configuration not only increases redundancy but also allows for the acquisition of the quadratic form of angular velocity, offering a new perspective for angular velocity computation [15]. Influenced by the six-accelerometer cubic configuration, early versions of the twelve-accelerometer configurations were all based on the cubic model. Xiao and her team innovated by merging two six-accelerometer systems to create an advanced twelve-accelerometer cube configuration [16]. Buhmann suggested organizing the twelve accelerometers into four triads, each positioned at the cube’s corners, designed to be responsive to three-dimensional force information [17]. Park proposed alternative twelve-accelerometer setups, including one with two accelerometers on each cube face and another with three accelerometers at four specific vertices [18]. In parallel with the cube configuration, various types of configuration schemes have emerged, notably one involving all twelve accelerometers aligned along the carrier’s axes, segmented into four triads. One triad is directly connected to the carrier’s centroid, while the other three are aligned along the positive axes, making each triad sensitive to three-dimensional force information [19]. This configuration can directly measure force at the carrier’s centroid and provide all the relevant quantities related to angular velocity, significantly improving the accuracy of angular velocity calculations. However, the primary challenge associated with the 12-accelerometer configuration scheme is that the angular velocity appears in a quadratic form within the system equations. In the presence of accelerometer noise, relying solely on the quadratic form of angular velocity is inadequate for determining its direction, necessitating the use of additional methods to address this issue. Wang et al. designed a twelve-accelerometer configuration scheme on a bullet’s body, derived the square term of angular velocity, used the integral algorithm of angular acceleration to determine positive and negative signs, extracted the square term, and combined it with specific filtering links to obtain the output of angular velocity [20]. A method proposed in [21] uses redundant information to obtain the residual error equation, followed by numerical iteration to enhance accuracy, but this method is only suitable for short-term attitude calculation. Wang suggested a fusion algorithm that integrates the integral algorithm and extraction algorithm to calculate angular velocity based on a nine-accelerometer configuration scheme; however, this approach fails to address the extraction algorithm’s sign misjudgment problem fundamentally [22].

Based on the aforementioned research findings, this paper introduces a new algorithm for calculating angular velocity using Kalman filtering, aiming to enhance accuracy. The proposed method effectively addresses the issue of sign misjudgment in the extraction algorithm by incorporating the sliding window technique, thereby improving the accuracy of the collected data. In this approach, the angular velocity obtained through the integral algorithm is considered as the state variable, while the angular velocity derived from the improved extraction algorithm is used as the observation value. These two sets of angular velocity data are then combined using Kalman filtering. Finally, the fused data are fed into quaternionic equations to determine the instrument’s attitude change. This comprehensive approach significantly contributes to the enhancement of angle calculation accuracy.

## 2. Fundamentals

### 2.1. GFSINS Principles

**Figure 1:**
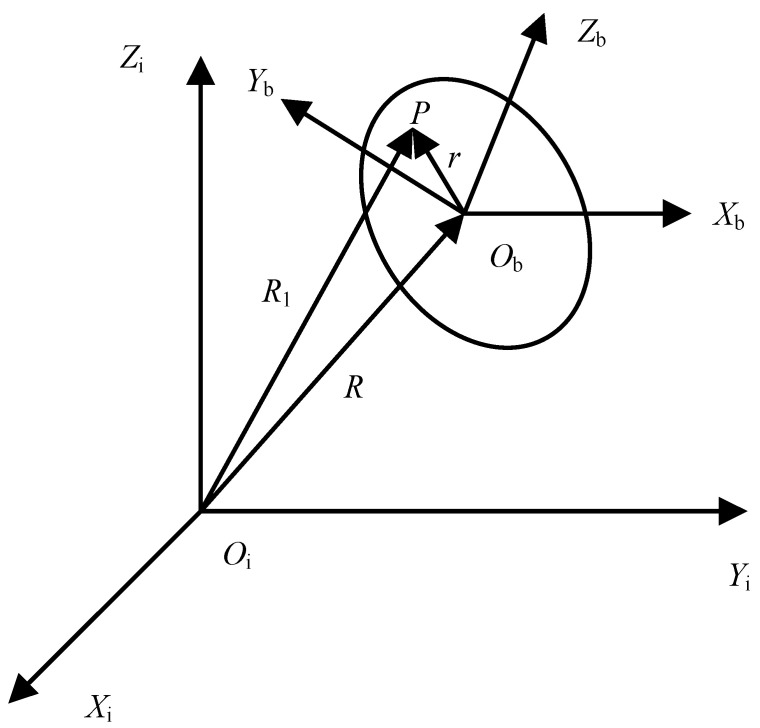
Vector diagram between inertial frame and body frame.

The full accelerometer inertial guidance system, commonly referred to as gyro-free inertial navigation system (GFSINS), utilizes accelerometers placed at the non-center of mass of the carrier to detect the angular motion of the body. This process enables the calculation of the specific force and angular velocity at the center of mass of the body using the output of the accelerometers. The inertial frame is defined as Oi-XiYiZi, while the body frame, where the accelerometer array is arranged, is denoted as Ob-XbYbZb (see Figure 1). R is a position vector from the center of the inertial frame to the center of the body frame and r is a position vector from the center of the body frame to any point P on the body. Consequently, the position vector $R_{1}$, representing the distance from the center of the inertial frame to the non-center of mass of the body, is expressed in the following figure.

(1) $$R_{1}=R+r$$

The formula to calculate the absolute acceleration of any point *P* on the body frame is as follows:

(2) $$\frac{d_{i}^{2}Ri}{dt^{2}}=\frac{d_{i}^{2}R}{dt^{2}}+\dot{\omega }\times r+\omega \times \left(\omega \times r\right).$$

By installing n accelerometers on the body according to a specific configuration, where the installation position of the *i*th accelerometer is $L_{i}$ and its sensitive direction is $\theta_{i}$, the expression for the accelerometer’s output in terms of specific force is as follows:

(3) $$f_{i}=\left[{\left(u_{i}\times \theta_{i}\right)}^{T}\theta_{i}\right]\times \left[\begin{array}{c} \dot{\omega }\\ A \end{array}\right]+\theta_{i}^{T}\Omega^{2}L_{i}.$$

In (3), $\Omega =\left[\begin{array}{ccc} 0 & -\omega_{z} & \omega_{y}\\ \omega_{x} & 0 & -\omega_{z}\\ -\omega_{y} & \omega_{x} & 0 \end{array}\right]$, $\dot{\omega }=\left[\begin{array}{c} {\dot{\omega }}_{x}\\ {\dot{\omega }}_{y}\\ {\dot{\omega }}_{z} \end{array}\right]$, $A=\left[\begin{array}{c} A_{x}\\ A_{y}\\ A_{z} \end{array}\right]$. $A_{x}$, $A_{y}$, and $A_{z}$ represent the acceleration of the body frame *b* with respect to the inertial frame *i* along the three axes of the body frame.

### 2.2. Configuration Scheme

**Figure 2:**
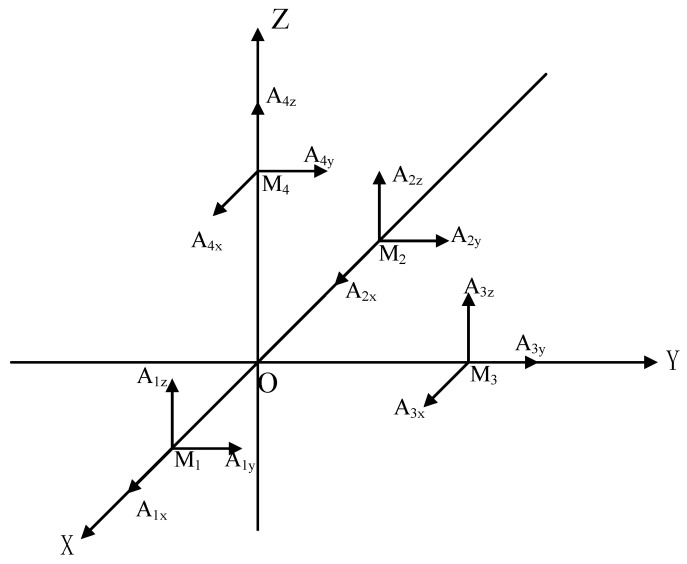
Schematic diagram of twelve-accelerometer array configuration scheme.

By incorporating six linear accelerometers in the body frame, it becomes possible to derive the angular velocity of each axis within the system by solving the accelerometer output. This information can then be utilized for attitude calculation. In this study, we have employed a twelve-accelerometer configuration scheme consisting of four three-axis accelerometers. This configuration scheme utilizes redundancy to compensate for system errors, thereby enhancing the overall accuracy. Furthermore, it ensures that the sensitive directions of the accelerometers mounted along the same axes are mutually orthogonal, making it well suited for installation in narrow spaces [23]. The specific arrangement of the twelve-accelerometer array is depicted in Figure 2, where M_1_~M_4_ represent the accelerometer mounting points, and A_1x_~A_4z_ denote the mounting directions of the corresponding sensitive axes. For more information on the specific mounting position and sensitive directions of the twelve-accelerometer array, please refer to Table 1.

Let $A_{1x}$, $A_{1y}$, $A_{1z}$, …, $A_{4z}$ represent the output acceleration values from the accelerometers. By substituting L, θ into Equation (3), we can establish the relationship between the motion parameters of the carrier and the measured acceleration value as follows:

(4) $$\left\{\begin{array}{c} {\ddot{R}}_{x}=(A_{1x}+A_{2x})/2\\ {\ddot{R}}_{y}=(A_{1y}+A_{2y})/2\\ {\ddot{R}}_{z}=(A_{1z}+A_{2z})/2 \end{array}\right.,$$

(5) $$\left\{\begin{array}{c} {\dot{\omega }}_{x}=\left(A_{1y}+A_{2y}+2A_{3z}-A_{1z}-A_{2z}-2A_{4y}\right)/4l\\ {\dot{\omega }}_{y}=\left(A_{2z}+2A_{4x}-A_{1x}-A_{1z}-A_{2x}\right)/4l\\ {\dot{\omega }}_{z}=\left(A_{1x}+A_{1y}+A_{2x}-A_{2y}-2A_{3x}\right)/4l \end{array}\right.,$$

(6) $$\left\{\begin{array}{c} \omega_{x}^{2}=\left(A_{1x}+A_{1y}+A_{2y}+A_{1z}-A_{2x}+A_{2z}-2A_{3y}-2A_{4z}\right)/4l\\ \omega_{y}^{2}=\left(A_{2x}+2A_{3y}+A_{1z}+A_{2z}-A_{1x}-A_{1y}-A_{2y}-2A_{4z}\right)/4l\\ \omega_{z}^{2}=\left(A_{2x}+A_{1y}+A_{2y}+2A_{4z}-A_{1x}-2A_{3y}-A_{1z}-A_{2z}\right)/4l \end{array}\right..$$

## 3. Attitude Algorithm

### 3.1. Angular Velocity Calculation

The primary approaches for calculating angular velocity based on the terms associated with angular acceleration and angular velocity are primarily through the integral algorithm and extraction algorithm [24]. These methods are outlined as follows:

- Integral algorithm:

Direct integration of Equation (5) yields an expression for the angular velocity of the motion around the carrier coordinate axis as

(7) $$\omega \left(t+T\right)=\omega \left(t\right)+\dot{\omega }\left(t\right)T.$$

Here, t represents the sampling instant, T denotes the sampling period, and $\omega \left(t+T\right)$ signifies the angular velocity obtained through integration at the subsequent moment.

- Integral algorithm:

The absolute value of the angular velocity is obtained by extracting the square root of the term in Equation (6) representing the squared angular velocity. The sign of the angular velocity is then determined from the result obtained by integrating Equation (8).

To determine the absolute value of the angular velocity, we square the term in Equation (6) representing the angular velocity squared. The sign of the angular velocity, on the other hand, is determined by Equation (7), and examining the resulting outcome:

(8) $$\omega \left(t\right)=sign\left(\omega \left(t\right)\right)\cdot \left|\sqrt{\omega {\left(t\right)}^{2}}\right|.$$

The output of the accelerometer is prone to errors caused by installation bias, scale factor, zero bias, and random noise. When using the integral method for solving, these errors are transferred to the calculation of angular velocity at subsequent moments. Over time, as the test duration increases, these errors accumulate and result in a decrease in the accuracy of the angular velocity solution. On the other hand, the extraction algorithm can avoid integration errors. However, the reliability of the sign value obtained through the integral method diminishes over time, leading to sign misclassification near zero values of angular velocity. This misclassification of signs adversely affects the accuracy of the angular velocity calculation. Through analysis, it has been observed that this sign miscalculation manifests as the localized appearance of extreme values in the angular velocity curve, with opposite signs of angular velocity between two adjacent data points at these extreme value locations.

### 3.2. Improved Extraction Algorithm

To effectively address the occurrence of extreme values and sign changes in eliminating outliers from the traditional extraction algorithm’s calculated results, this study employs the sliding window method to manipulate the data series. This approach is characterized by its high computational efficiency while still maintaining the temporal characteristics of the data [25,26,27]. The following are the specific processing steps:

- Determining the sliding window size.

To identify outliers accurately, it is crucial to select a suitable window size that includes the outlier point and its adjacent points. Through several experiments conducted in this study, it has been determined that an optimal window size of 3 effectively detects the presence of outliers.

- Defining the Window Function.

In order to tackle the issue of anomalies frequently occurring within a narrow interval, with neighboring points exhibiting opposite signs, we introduce a window characteristic function:

(9) $$Y=\max{\left\{\left(\omega_{t}-\omega_{t-1}\right)\left(\omega_{t+1}-\omega_{t}\right),\omega_{t-1}\omega_{t+1}\right\}}_{i}.$$

By utilizing this window function, anomalies within the data set can be identified more efficiently, and noise can be eliminated. In this context, $\omega_{t-1}$, $\omega_{t}$, and $\omega_{t+1}$ represent the three angular velocity values within the *i*th window. Whenever an anomaly exists within the *i*th window, the value of the window’s characteristic function is negative.

- Detecting Anomalies.

The feature function is calculated for each window, and upon obtaining a negative value for the characteristic function, the center point of the window can be identified as an anomaly.

- Correcting Angular Velocity Sign.

The position of the anomaly is recorded, and the corresponding value’s sign is reversed to obtain the angular velocity result after sliding window processing.

### 3.3. Kalman Filter Fusion Algorithm

**Figure 3:**
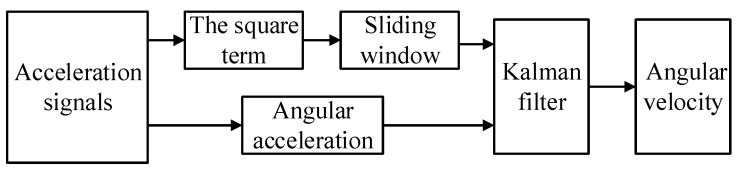
Schematic diagram of angular velocity fusion.

Wang et al. conducted a study using Kalman filtering to analyze random signals in vibration testing. The results demonstrated that compared to a single-calculation algorithm, Kalman filtering significantly reduces the maximum value and standard deviation of errors while also improving the accuracy of angular velocity amplitude and the smoothness of curves [28,29]. Building upon this existing algorithm, this paper utilizes Kalman filtering to fuse data from both the integral algorithm and the improved extraction algorithm. Specifically, the angular velocity calculated by the integral algorithm is employed as the state variable, while the angular velocity calculated by the improved extraction algorithm serves as the observation value. The fusion process is illustrated in Figure 3.

The Kalman filter-based angular velocity fusion algorithm is implemented as follows:

- Discretizing Equation (7), the system’s state prediction equation is obtained:

(10) $$X_{k}=X_{k}+u_{k-1}+W_{k},$$

where $X_{k}={\left[\begin{array}{ccc} \omega_{x} & \omega_{y} & \omega_{z} \end{array}\right]}^{T}$ is the angular velocity of rotation around the three axes, $W_{k}$ is the noise of the unknown system, and its covariance matrix is $Q_{k}$; $u_{k-1}$ is the state transition control term, expressed as

(11) $$u_{k-1}={\left[\begin{array}{ccc} {\dot{\omega }}_{x} & {\dot{\omega }}_{y} & {\dot{\omega }}_{z} \end{array}\right]}^{T}{|}_{k-1}\cdot T.$$

- The observation equation for the system is established as follows:

(12) $$Z_{k}=H_{k}X_{k}+V_{k},$$

where $H_{k}X_{k}$ is the improved extraction algorithm value and $V_{k}$ is the observation noise with covariance matrix R.

- Update the state variable estimates:

(13) $${\hat{X}}_{k}={\hat{X}}_{k/k-1}+K_{k}\left[Z_{k}-H_{k}{\hat{X}}_{k/k-1}\right].$$

- Predict the error covariance array:

(14) $$P_{k/k-1}=AP_{k-1}A^{T}+Q_{k}.$$

- Calculate the Kalman gain matrix:

(15) $$K_{k}=P_{k/k-1}H_{k}^{T}{\left[H_{k}P_{k/k-1}H_{k}^{T}+R_{k}\right]}^{-1}.$$

- Update the error covariance array:

(16) $$P_{k}=\left(I-K_{k}H_{k}\right)P_{k/k-1}.$$

By continuously repeating the steps of prediction and updating, the optimal estimates of the state variables at each moment can be computed in real time to obtain ${\hat{X}}_{k}$, which represents the best estimate of the angular velocity.

### 3.4. Fuaternion Mean

Given the continuous changes in the carrier’s attitude angle, it is crucial to promptly update the attitude matrix. The commonly used methods for updating include the Euler angle method, direction cosine method, and quaternion method. However, the direction cosine method requires a significant amount of computation, while the Euler angle method involves heavy computation and is susceptible to backstepping issues [30,31,32]. Taking these considerations into account, this paper adopts the quaternion method for updating the attitude matrix. We define $Q={\left[\begin{array}{cccc} q_{0} & q_{1} & q_{2} & q_{3} \end{array}\right]}^{T}$ as the rotated quaternion representation of the laser scanning projection system’s coordinate system at a particular sampling moment relative to the previous sampling moment’s coordinate system. We can then obtain the expression of the attitude matrix using this definition:

(17) $$R=\left[\begin{array}{ccc} q_{0}^{2}+q_{1}^{2}-q_{2}^{2}-q_{3}^{2} & 2(q_{1}q_{2}-q_{0}q_{3}) & 2(q_{1}q_{3}+q_{0}q_{2})\\ 2(q_{1}q_{2}+q_{0}q_{3}) & q_{0}^{2}-q_{1}^{2}+q_{2}^{2}-q_{3}^{2} & 2(q_{2}q_{3}-q_{0}q_{1})\\ 2(q_{1}q_{3}-q_{0}q_{2}) & 2(q_{2}q_{3}+q_{0}q_{1}) & q_{0}^{2}-q_{1}^{2}-q_{2}^{2}+q_{3}^{2} \end{array}\right].$$

By applying the carrier coordinate system and the geographic coordinate system definitions, it is possible to derive quadratic differential equations that describe the correlation between angular rate and angular position.

(18) $$\frac{dQ}{dt}=\frac{1}{2}Q\cdot {\dot{\omega }}_{Eb}^{b}$$

Expanding further, we have

(19) $$\begin{array}{c} \frac{dQ}{dt}=\frac{1}{2}\left(q_{0}+q_{1}\hat{i}+q_{2}\hat{j}+q_{3}\hat{k}\right)\cdot \left(\omega_{x}\hat{i}+\omega_{y}\hat{j}+\omega_{z}\hat{k}\right)\\ =\frac{1}{2}\left[\begin{array}{cccc} 0 & \omega_{x} & -\omega_{y} & -\omega_{z}\\ \omega_{x} & 0 & \omega_{z} & -\omega_{y}\\ \omega_{y} & -\omega_{z} & 0 & \omega_{x}\\ \omega_{z} & \omega_{y} & -\omega_{x} & 0 \end{array}\right]\left[\begin{array}{c} q0\\ q1\\ q2\\ q3 \end{array}\right] \end{array}.$$

By employing the first-order Lungkuta method to solve the aforementioned equation, we obtain real-time updated expressions for quaternions and attitude angles:

(20) $${\left(\begin{array}{c} q_{0}\\ q_{1}\\ q_{2}\\ q_{3} \end{array}\right)}_{t+\Delta t}={\left(\begin{array}{c} q_{0}\\ q_{1}\\ q_{2}\\ q_{3} \end{array}\right)}_{t}+\frac{\Delta t}{2}\left(\begin{array}{c} -\omega_{x}\cdot q_{1}-\omega_{y}\cdot q_{2}-\omega_{z}\cdot q_{3}\\ \omega_{x}\cdot q_{0}-\omega_{y}\cdot q_{3}+\omega_{z}\cdot q_{2}\\ \omega_{x}\cdot q_{3}+\omega_{y}\cdot q_{0}-\omega_{z}\cdot q_{1}\\ -\omega_{x}\cdot q_{2}+\omega_{y}\cdot q_{1}+\omega_{z}\cdot q_{0} \end{array}\right).$$

Here, the variables ϕ, θ, and ψ represent the roll, pitch, and heading angles of the laser scanning projection system, respectively.

(21) $$\left[\begin{array}{c} \phi \\ \theta \\ \psi \end{array}\right]=\left[\begin{array}{c} \mathrm{atan}2(2(q_{0}q_{1}+q_{2}q_{3}),1-2(q_{1}^{2}+q_{2}^{2}))\\ a\sin(2(q_{0}q_{2}-q_{3}q_{1}))\\ \mathrm{atan}2(2(q_{0}q_{3}+q_{1}q_{2}),1-2(q_{2}^{2}+q_{3}^{2})) \end{array}\right]$$

## 4. Simulation

To validate the efficacy of the proposed method for solving angular velocity, simulation experiments were conducted in this study using MATLAB R2020a. The following mathematical model of angular velocity was utilized during the simulation process (where $\omega_{m}$ is 0.25 rad/s):

(22) $$\left\{\begin{array}{c} \omega_{x}=2\omega_{m}\sin(10t)\\ \omega_{y}=4\omega_{m}\cos(20t)\\ \omega_{z}=\omega_{m}\sin(t)+t^{2} \end{array}\right..$$

For a practical value of the accelerometer’s noise and constant value error, we consider the specifications of the accelerometers manufactured by TDK Corporation. The simulation parameters are set as follows: a sampling frequency of 500 Hz, a sampling time of 10 s, constant value error of the accelerometer at 15 μg, mean square deviation of random noise at 9 μg/√Hz, and an accelerometer mounting position at a distance of L = 0.04 m from the center of the carrier coordinates.

### 4.1. Simulation of the Basic Algorithm

**Figure 4:**
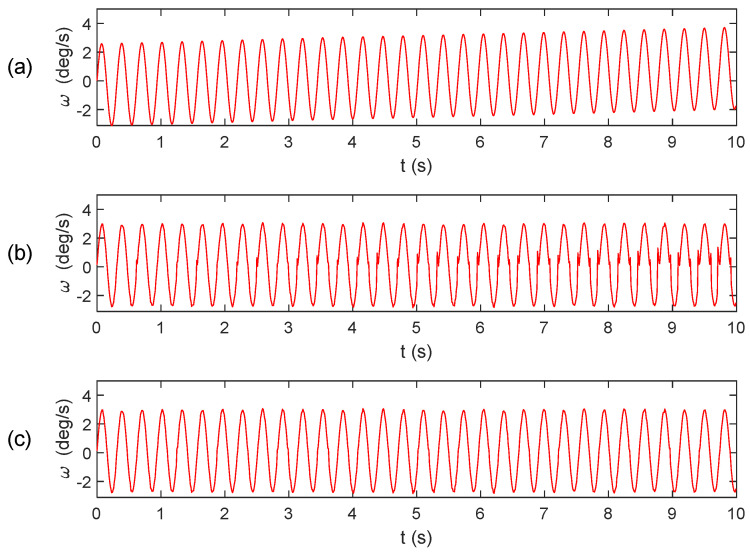
Angular velocity calculation results: (**a**) integral algorithm; (**b**) extraction algorithm; (**c**) improved extraction algorithm.

**Figure 5:**
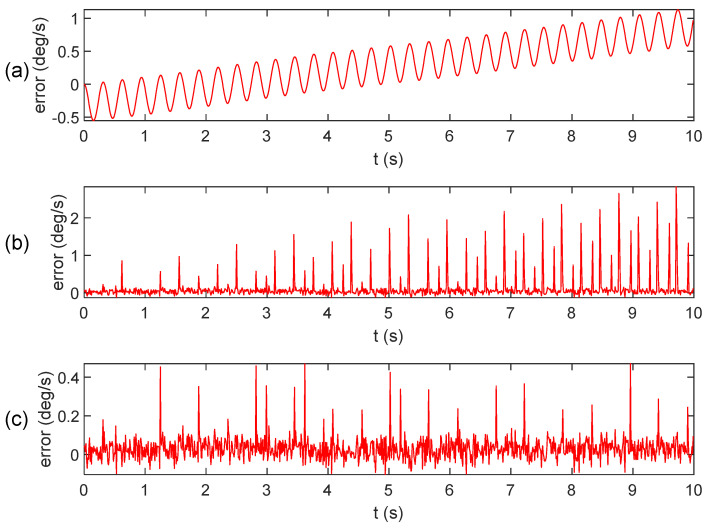
Angular velocity calculation error: (**a**) integral algorithm; (**b**) extraction algorithm; (**c**) improved extraction algorithm.

Figure 4 and Figure 5 display the results of calculating the angular velocity using the integral algorithm, extraction algorithm, and improved extraction algorithm, along with their respective error.

From Figure 5, it is evident that the angular velocity error in the integral algorithm gradually accumulates over time. The extraction algorithm exhibits a sign misclassification phenomenon. However, the improved extraction algorithm effectively mitigates the abnormal bulge near the zero value and results in a smoother curve.

### 4.2. The Fusion Algorithm

**Figure 6:**
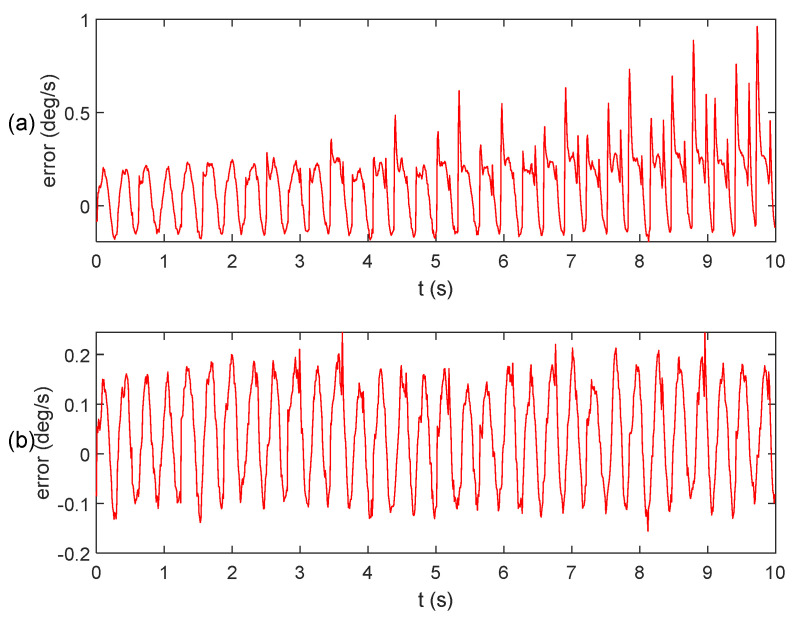
Angular velocity calculation error: (**a**) existing algorithm; (**b**) our algorithm.

The existing algorithm involves fusing data from the integral algorithm and the extraction algorithm. This section presents a comparative analysis of the results between the proposed algorithm and the existing algorithm, demonstrating the computational errors as shown in Figure 6.

**Figure 7:**
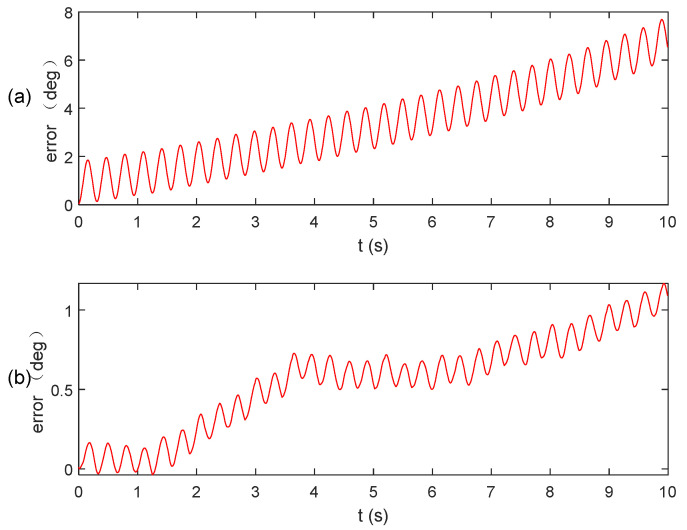
Angular calculation error: (**a**) existing algorithm; (**b**) our algorithm.

Subsequently, the results obtained from the two aforementioned algorithms are input into the quaternion equation for calculation, and the resulting angular calculation error is presented in Figure 7. Notably, the algorithm proposed in this paper exhibits significantly higher solution accuracy compared to the existing algorithm. This improvement can be attributed to the utilization of the sliding window method, which effectively eliminates anomalies near the zero value.

To provide a more precise depiction of the superiority of the algorithm proposed in this paper, statistical data on the parameter error of the two algorithms are presented in Table 2. It is evident from Table 2 that the accuracy of attitude calculation achieved by the fused integral algorithm and the improved extraction algorithm proposed in this paper surpasses that of the existing algorithm, with a reduction in accuracy of one order of magnitude for both the maximum error and standard deviation. Therefore, it can be assumed that this algorithm is capable of accurately solving attitude-related problems for relevant instruments even under vibration conditions.

## 5. Experiment

**Figure 8:**
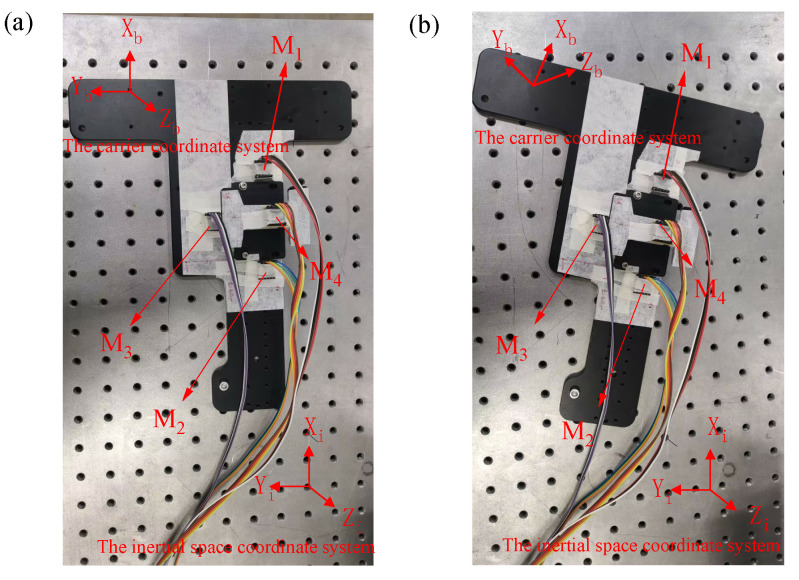
Position of the experimental board (**a**) before the experiment and (**b**) after the experiment.

To validate the feasibility of the proposed algorithm, an experimental board with four three-axis accelerometers was placed on an optical platform. The installation positions of the accelerometers, labeled as M_1_ to M_4_, are illustrated in Figure 8. External jitters and impacts were manually applied to test the board. The accelerometer utilizes the AXO301 accelerometer produced by TDK Corporation. The primary specifications are shown in Table 3.

**Figure 9:**
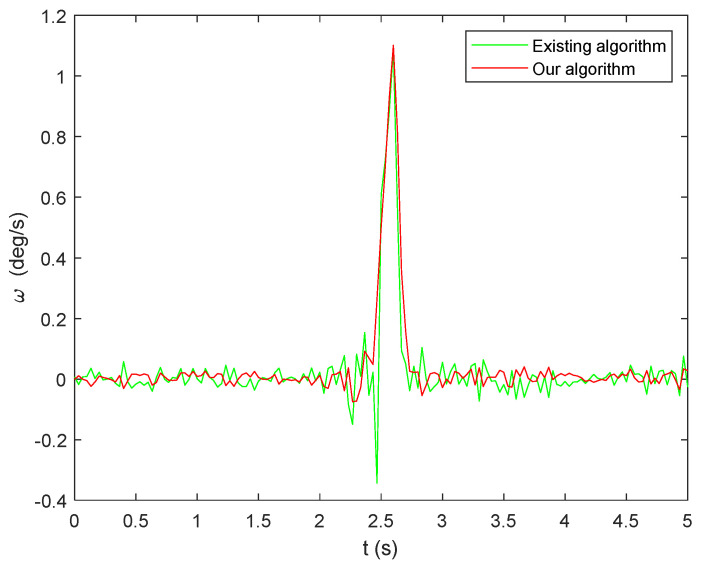
Angular velocity calculation results.

Throughout the experiment, a sampling frequency of 100 Hz was utilized, and a total of 12 sets of accelerometer data were collected. The proposed algorithm as well as the existing algorithm were both applied to calculate the angular velocity, taking the *Z*-axis as an example. The outcomes are depicted in Figure 9.

**Figure 10:**
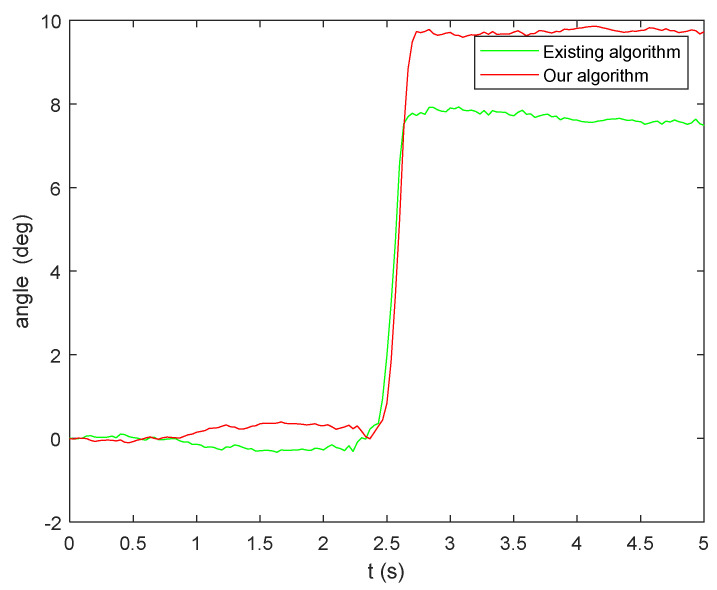
Angle calculation results.

**Figure 11:**
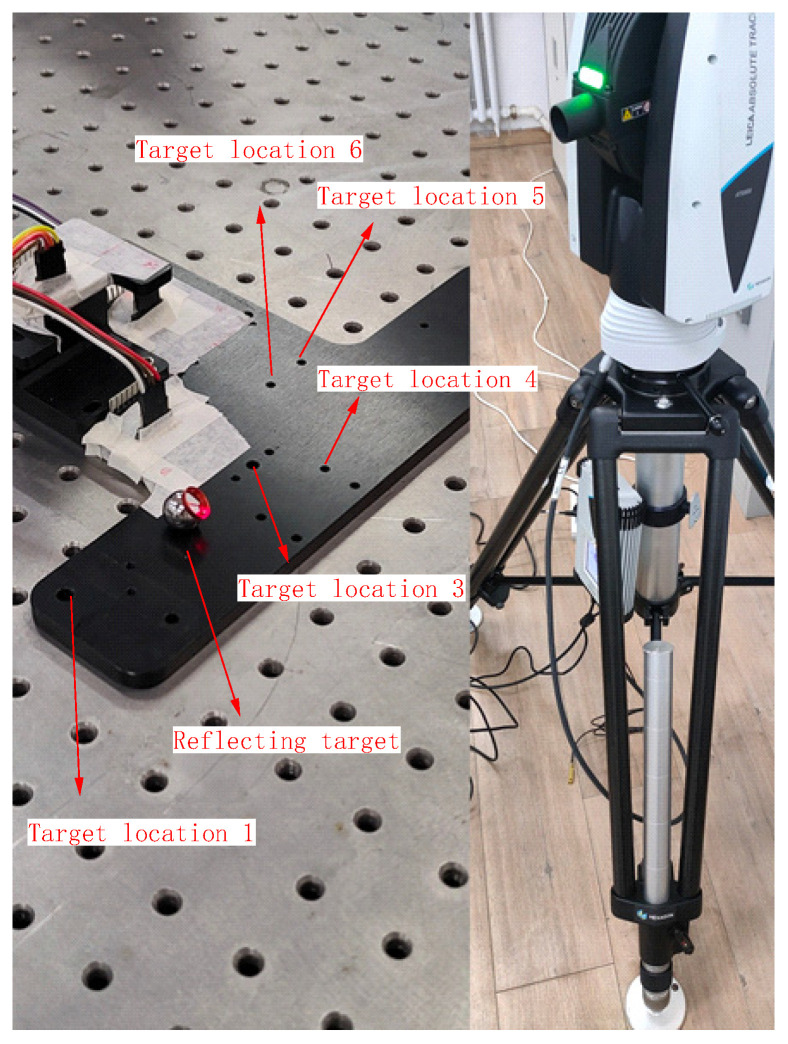
Verification of attitude calculation accuracy in accelerometer array through a tracker.

To facilitate a more intuitive comparison of the accuracy among the aforementioned algorithm, the angular velocity results were input into the quaternion equation to calculate the angular change, as shown in Figure 10. The accuracy of the accelerometer array attitude calculation was then verified using a laser tracker (see Figure 11).

Figure 10 clearly demonstrates that the existing algorithm yields an inaccurate estimate, which is subsequently used as input in the quaternion equation. This inaccuracy becomes more pronounced when observing the magnitude of the angular curve, mainly due to the unresolved sign misclassification problem associated with the extraction algorithm. However, when combining the integral algorithm and the improved extraction algorithm, the resulting angle solution curve appears smoother.

Table 4 presents the corresponding rotation matrices and angle changes for both methods.

The experiment was conducted five times to compare the results of the accelerometer array attitude solving with the laser tracker solving. The comparison is presented below, using the *Z*-axis as an example.

Table 5 illustrates that the Kalman filter-based angular velocity fusion algorithm proposed in this paper yields highly accurate results. The proposed algorithm reduces the error by one order of magnitude when compared to existing methods, with an average difference between the solution angle and the angle measured by the laser tracker of less than 0.5°. This level of accuracy meets the necessary requirements for measuring attitude change in the laser scanning projection system.

## 6. Conclusions

The present study introduces an angular velocity fusion algorithm based on Kalman filtering. To address the issue of sign misjudgment in the open method for solving angular velocity, we propose a sliding window correction method that enhances the accuracy of this approach. By combining data from the integral method and the improved open method, we leverage Kalman filtering to obtain the optimal estimate of angular velocity. Simulation experiments validate the feasibility and effectiveness of our proposed algorithm. Specifically, the results demonstrate that the maximum value and standard deviation of angular velocity error can be reduced by one order of magnitude compared to existing algorithms while also improving accuracy and curve smoothness. Furthermore, experiments confirm that our algorithm exhibits superior attitude-solving accuracy relative to existing approaches. Overall, this study presents a practical, accurate, and straightforward solution for detecting instrument attitude under actual working conditions, with significant application value.

## Figures and Tables

**Table 1:** Twelve-accelerometer mounting positions and directions.

Accelerometer Serial Number	Installation Position L = [Lx, Ly, Lz]	Sensitive Direction θ = [θx, θy, θz]
M_1_ (A_1x_, A_1y_, A_1z_)	[l, 0, 0]	[1 0 0; 0 1 0; 0 0 1]
M_2_ (A_2x_, A_2y_, A_2z_)	[−l, 0, 0]	[1 0 0; 0 1 0; 0 0 1]
M_3_ (A_3x_, A_3y_, A_3z_)	[0, l, 0]	[1 0 0; 0 1 0; 0 0 1]
M_4_ (A_4x_, A_4y_, A_4z_)	[0, 0, l]	[1 0 0; 0 1 0; 0 0 1]

**Table 2:** Statistics of the angular calculation errors.

Method	Maximum Absolute Error	Average Relative Error	Standard Deviation
Existing algorithm	7.8823	3.4295	1.8103
Our algorithm	1.1435	0.5534	0.2978

**Table 3:** The primary parameters of AXO301 accelerometer.

Parameter (Unit)	Input Range (g)	Digital Resolution (µg/LSB)	Noise Density (μg/√Hz)	Bias Instability (μg)	Constant Bias (mg)
Value	±1	1	9	3	0.5

**Table 4:** Rotation matrix and angle change value.

Method	Rotation Matrix	Angle Change/(°)
Existing algorithm	$\left[\begin{array}{ccc} 0.9941 & -0.1080 & 0.0067\\ 0.1079 & 0.9941 & -0.0142\\ -0.0082 & -0.0134 & 0.9999 \end{array}\right]$	$\left[\begin{array}{ccc} -0.9648 & 0.5197 & 7.6893 \end{array}\right]$
Our algorithm	$\left[\begin{array}{ccc} 0.9861 & -0.1655 & 0.0102\\ 0.1656 & 0.9861 & -0.0054\\ -0.0091 & 0.0070 & 0.9999 \end{array}\right]$	$\left[\begin{array}{ccc} 0.2118 & 0.0057 & 9.6705 \end{array}\right]$

**Table 5:** Angle error values measured by two algorithms.

**Experiment Number**	**Angle Measured by Laser Tracker**	**Existing Algorithm**	**Our Algorithm**	**Minimum Absolute Error**
1	10.1317	7.6893	9.6705	0.4612
2	7.4615	8.1204	7.1427	0.3188
3	11.0265	8.6521	10.5531	0.4734
4	−4.3621	−6.2212	−4.0082	0.3539
5	−7.4342	−6.1290	−6.9908	0.4434

## Data Availability

Data are contained within the article.
